# A general and efficient method for estimating continuous IBD functions for use in genome scans for QTL

**DOI:** 10.1186/1471-2105-8-440

**Published:** 2007-11-13

**Authors:** Francois Besnier, Örjan Carlborg

**Affiliations:** 1 Linnaeus Centre for Bioinformatics, Uppsala University, SE-75124 Uppsala, Sweden; 2 Department of Animal Breeding and Genetics, Swedish University of Agricultural Sciences, SE-75005 Uppsala, Sweden

## Abstract

**Background:**

Identity by descent (IBD) matrix estimation is a central component in mapping of Quantitative Trait Loci (QTL) using variance component models. A large number of algorithms have been developed for estimation of IBD between individuals in populations at discrete locations in the genome for use in genome scans to detect QTL affecting various traits of interest in experimental animal, human and agricultural pedigrees. Here, we propose a new approach to estimate IBD as continuous functions rather than as discrete values.

**Results:**

Estimation of IBD functions improved the computational efficiency and memory usage in genome scanning for QTL. We have explored two approaches to obtain continuous marker-bracket IBD-functions. By re-implementing an existing and fast deterministic IBD-estimation method, we show that this approach results in IBD functions that produces the exact same IBD as the original algorithm, but with a greater than 2-fold improvement of the computational efficiency and a considerably lower memory requirement for storing the resulting genome-wide IBD. By developing a general IBD function approximation algorithm, we show that it is possible to estimate marker-bracket IBD functions from IBD matrices estimated at marker locations by any existing IBD estimation algorithm. The general algorithm provides approximations that lead to QTL variance component estimates that even in worst-case scenarios are very similar to the true values. The approach of storing IBD as polynomial IBD-function was also shown to reduce the amount of memory required in genome scans for QTL.

**Conclusion:**

In addition to direct improvements in computational and memory efficiency, estimation of IBD-functions is a fundamental step needed to develop and implement new efficient optimization algorithms for high precision localization of QTL. Here, we discuss and test two approaches for estimating IBD functions based on existing IBD estimation algorithms. Our approaches provide immediately useful techniques for use in single QTL analyses in the variance component QTL mapping framework. They will, however, be particularly useful in genome scans for multiple interacting QTL, where the improvements in both computational and memory efficiency are the key for successful development of efficient optimization algorithms to allow widespread use of this methodology.

## Background

Variance component analysis [1] is a flexible strategy for detecting Quantitative Trait Loci (QTLs), which is particularly useful in general populations such as humans and livestock. In the variance component framework, the QTL effect of each individual in the population is modelled as a random effect. The covariance structure of this random effect is proportional to the probability of identity by descent (IBD) at the location in the genome tested for QTL. The covariance matrix, also referred to as the IBD matrix, contains the probabilities for each pair of individuals in the studied sample sharing an allele identical by descent (i.e. the probability that a particular allele has been inherited from a common ancestor in the base population). The estimation of the IBD matrix from pedigree and genetic marker information is thus a central, and often very time-consuming, component of variance component QTL analyses.

Several methods have been proposed to estimate IBD matrices in a wide range of population structures. [2-5] Some methods are deterministic [2] and other stochastic, based on simulation [3], but all use information from genetic markers and pedigree. In QTL mapping, these methods are used to compute IBD matrices at pre-defined locations, a grid, in the genome where variance components for QTL will subsequently be estimated. Due to the computational demand in computing IBD matrices and estimating QTL variance components, plus the large memory requirement for storing large numbers of IBD matrices, it is common practice to restrict the resolution of the computational grid. Due to this, the optimal QTL location in the genome is not identified and consequently the power to detect QTL is not maximised. Global optimization algorithms [6,7] have been shown to be a computationally efficient approach to search the genomic grid where the genetic relationships have been estimated in a least square based QTL mapping framework [8] In the least squares framework, the cost of computing and storing QTL genotype probabilities at high resolution is negligible compared to the statistical estimation of genetic effects. In variance component QTL mapping on the other hand, the high computational cost both in estimating variance components and computing and storing IBD matrices indicate that new and efficient algorithms for computing and storing IBD as well as for estimation of variance components are needed to facilitate more in-depth exploratory analyses of experimental data using variance component models.

We have recently reported an efficient method for variance component estimation in QTL mapping [9]. Here, we propose a new approach for genome-wide IBD estimation in pedigrees. Rather than using the currently predominating approach, which estimates discrete IBD's at pre-defined genomic locations, we estimate genome-wide IBD as a series of continuous marker interval IBD-functions. With the current density of markers used in genome-wide QTL analysis (often 20–30 cM average marker spacing when using micro-satellites), more IBD matrices are required than the number of available markers. Consequently, the IBD probabilities are estimated not only at marker locations, but also at multiple locations in the interval between markers i.e interval mapping [10-12]. Since all the data required to estimate IBD probabilities comes from genetic markers and pedigree, the same data is used to compute all the IBD values within the interval between two consecutive markers (i.e. a marker bracket). Instead of predefining the locations in the marker bracket where IBD matrices should be calculated and then compute them independently, we show how to formulate the IBD between each pair of individuals as a function of the distance from the flanking marker positions. We then show how these IBD-functions, in the form of a single algebraic formulation, can be obtained by re-implementing existing IBD estimation algorithms or by estimating it from IBD matrices estimated at marker locations. The functions can then be used to cheaply calculate an IBD matrix at any location within the marker bracket. Functions to calculate IBD-matrices in brackets between markers have previously been described for a set of specific relationships [5,10], but those functions were used to produce discrete IBD matrices at pre-defined locations. Here, we generalize this concept for estimating the functions in general pedigrees and highlight the advantages using those functions as an output of the algorithm.

To illustrate the generality of the new approach to estimate IBD functions, we have worked out two algorithms for IBD-function estimation. First, we show one example of how to re-implement an existing IBD estimation algorithm [10] to compute IBD functions. Then, we develop a general algorithm to approximate IBD-functions using a small number of IBD-matrices from any existing IBD estimation method as input. The computational efficiency and memory requirement of both methods are assayed together with their precision to estimate IBD. Furthermore, we explore how the approximations affect variance component estimation in QTL mapping. We conclude by discussing how one by describing IBD as a continuous function provides the foundation for implementation of existing and development of new and efficient optimization algorithms for screening the genome for single and multiple QTL.

## Results

### Re-implementation of existing IBD estimation algorithms to obtain exact IBD functions

**Figure 1:**
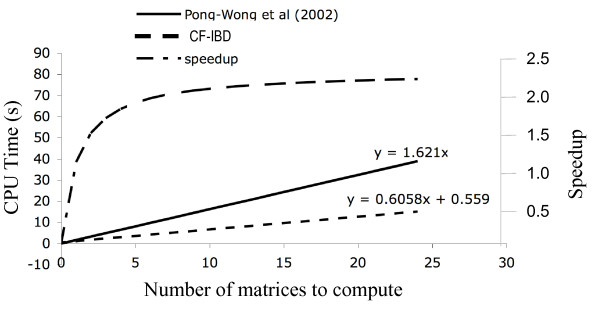
CPU time for computing increasing numbers of IBD matrices in a marker bracket using our updated algorithm based on IBD functions and Pong-Wong's original algorithm.

We re-implemented an algorithm for deterministic IBD matrix estimation [10] to compute marker-bracket IBD-functions instead of discrete IBD matrices. The new implementation produces IBD-matrices that are identical to those obtained using the original algorithm while at the same time improving the computational efficiency. Figure 1 compares the computational load imposed by our new implementation and the original algorithm for various number of IBD matrices calculated in a marker bracket. The new algorithm is always faster, and reaches a 2-fold speed-up when four matrices are estimated in a marker bracket and approaches a 2.6-fold speed-up asymptotically as the number of estimated matrices increases.

### A new and general algorithm for estimating IBD functions

We developed a general algorithm for approximating IBD-functions in marker brackets (CF-IBD – Continuous Function IBD) from a limited set of IBD matrices from any existing IBD estimation algorithm and evaluated how well it approximates the IBD matrices between markers that were obtained using the software LOKI [3].

This particular software was chosen because it is a stochastic algorithm based on Monte Carlo Markov Chain (MCMC) iteration procedure, which means that the computation strategy of this algorithm is completely different from the one of Ricardo Pong-Wong's method. By using this method to illustrate the efficiency of the curve-fitting approach, we also highlight the important aspect of the generality of this approach.

The input to our algorithm was IBD values at marker positions calculated by LOKI and the IBD matrices obtained from the IBD functions were compared to LOKI's IBD-matrices as they are the ones approximated by CF-IBD.

#### Precision of IBD matrices obtained from estimated IBD functions

**Figure 2:**
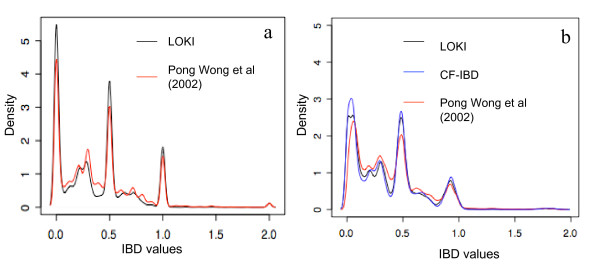
Empirical distribution function of IBD estimates in an 872 individual Jungelfowl × White Leghorn chicken F_2 _intercross at the marker *KITL *(a) and 5.4 cM from the marker (b) using the IBD estimation methods Pong-Wong et al (2002), LOKI and CF-IBD (only at the non-marker location in b).

In the optimal scenario, i.e. all markers are fully informative (the origin of all alleles are known), the IBD values are all either 0; 0.5; 1; (or 2 in the case of inbreeding). Consequently, a more informative IBD matrix has a higher density of those values than of intermediary values. None of the methods presented here requires fully informative markers, but we use the property mentioned above to postulate that the algorithm attribute the values 0, 0.5 or 1 when the IBD is estimated without uncertainty and that intermediate values then exist only when there is uncertainty. Our comparisons show that the IBD to be used for QTL mapping are likely to be much more influenced by the assumptions made when estimating IBD from data (i.e. in the IBD-estimation algorithm) rather than by the estimation of an IBD function from discrete IBD at marker locations. Figure 2 gives a graphical illustration of how informative the IBD-matrices obtained are from LOKI [3], CF-IBD when approximating LOKI IBD's, and from Pong-Wong's method [10]. The curve corresponding to CF-IBD is confounded with LOKI's IBD on Figure 2a, and is closer to LOKI's than to Pong-Wong's IBD on Figure 2b.

The distribution of the estimated IBDs were compared in a region close to marker KITL1 [13] located at position 141.2 cM on chicken chromosome 1 (Figure 2a) and in a second region 5.4 cM away from this same marker (Figure 2b). All three methods display a similar pattern of the individual IBD-values with a majority of values close to 0, 0.5 and 1 (for individuals with high marker information) and a range of intermediate values (for individuals with lower marker information). The estimates at the location away from the marker (Figure 2b) contain less information as shown by lower density of values close to 0, 0.5 and 1. LOKI and CF-IBD do, as expected, give very similar profiles, whereas the estimates using the algorithm of Pong Wong *et al*. produce less informative IBD matrices. To quantify the similarity between the methods, we have computed the average difference (i.e. the absolute pairwise difference between the non-null elements of two matrices) of the matrices computed by LOKI (L) to those of CF-IBD (CF) and the Pong-Wong (PW) based algorithm for several positions within the marker-bracket. Since the average expected value of the IBD between two individual is determined by their relationship (i.e.: 0.5 for full sibs or 0.25 for half-sibs), the pairwise differences have been weighted by this relationship coefficient. The differences between the methods are nearly constant within the marker brackets and is always bigger between PW and L, than between CF and L (weighted average difference of non-null elements in the matrices is 0.001 for CF vs L and 0.03 for PW vs L). Our approximations of LOKI are always closer to the original LOKI estimates than the estimates obtained using Pong-Wong's method, indicating that the effect of the assumptions used when calculating IBD at marker locations has a greater impact on the obtained IBD-matrices than the use of CF-IBD to approximate IBD in marker brackets.

#### Effects on estimated variance components

As the ultimate aim of estimating the IBD-matrices in QTL mapping applications is to use them for estimating genetic variance components of QTL, it is important to evaluate the potential decrease in statistical power from using an approximate IBD-estimation method rather than the exact method. Thus, we compared the variance component estimates obtained by REML [1] based on IBD matrices from LOKI estimates, CF-IBD approximations of LOKI IBDs and IBD estimates from the Pong-Wong algorithm in two regions on chicken chromosome 1, where QTL have previously been detected [13]. The use of CF-IBD approximations led to a slight under-estimation of the genetic variances – on average 98.7% and never smaller than 96% of the estimates obtained using LOKI IBDs. Estimation based on the IBD matrices obtained using the Pong-Wong algorithm were smaller with on average 81% and with a minimum of 58% of the LOKI based estimates.

#### Memory requirement for storing IBD matrices in a genome scan

Since our method considers the IBD values as a function of the recombination probability between two markers on the chromosome, it is possible to store the output of the algorithm as a function instead of a traditional IBD matrix. The IBD-function for each pair of individuals is calculated from a set of recombination functions (Table 1), and can be approximated numerically by a polynomial function that makes it easier to write and store in a file. Traditional IBD-matrices are normally sparse and stored accordingly with two id numbers and an IBD value for each pair of individuals with non-zero IBDs. The memory requirement for each matrix consequently depends on the number of non-zero elements in the matrix, and the numerical precision employed to store the result. The required memory to store a single matrix (*m*) can be calculated as:

**Table 1:** Probability Descent QTL (PDQ) functions for a marker interval to calculate the probability that a chromosomal segment is of paternal origin, given information in flanking markers.

Parental origin Marker 1	Parental origin Marker 2	Probability that QTL is of paternal origin
		
dam^1^	dam^1^	rarb1−r MathType@MTEF@5@5@+=feaafiart1ev1aaatCvAUfKttLearuWrP9MDH5MBPbIqV92AaeXatLxBI9gBaebbnrfifHhDYfgasaacPC6xNi=xH8viVGI8Gi=hEeeu0xXdbba9frFj0xb9qqpG0dXdb9aspeI8k8fiI+fsY=rqGqVepae9pg0db9vqaiVgFr0xfr=xfr=xc9adbaqaaeGacaGaaiaabeqaaeqabiWaaaGcbaqcfayaamaalaaabaGaemOCai3aaSbaaeaacqWGHbqyaeqaaiabdkhaYnaaBaaabaGaemOyaigabeaaaeaacqqGXaqmcqGHsislcqWGYbGCaaaaaaa@3569@
sire^1^	sire^1^	(1−ra)(1−rb)1−r MathType@MTEF@5@5@+=feaafiart1ev1aaatCvAUfKttLearuWrP9MDH5MBPbIqV92AaeXatLxBI9gBaebbnrfifHhDYfgasaacPC6xNi=xH8viVGI8Gi=hEeeu0xXdbba9frFj0xb9qqpG0dXdb9aspeI8k8fiI+fsY=rqGqVepae9pg0db9vqaiVgFr0xfr=xfr=xc9adbaqaaeGacaGaaiaabeqaaeqabiWaaaGcbaqcfa4aaSaaaeaacqGGOaakcqaIXaqmcqGHsislcqWGYbGCdaWgaaqaaiabdggaHbqabaGaeiykaKIaeiikaGIaeGymaeJaeyOeI0IaemOCai3aaSbaaeaacqWGIbGyaeqaaiabcMcaPaqaaiabigdaXiabgkHiTiabdkhaYbaaaaa@3C8D@
dam^1^	sire^1^	(ra)(1−rb)r MathType@MTEF@5@5@+=feaafiart1ev1aaatCvAUfKttLearuWrP9MDH5MBPbIqV92AaeXatLxBI9gBaebbnrfifHhDYfgasaacPC6xNi=xH8viVGI8Gi=hEeeu0xXdbba9frFj0xb9qqpG0dXdb9aspeI8k8fiI+fsY=rqGqVepae9pg0db9vqaiVgFr0xfr=xfr=xc9adbaqaaeGacaGaaiaabeqaaeqabiWaaaGcbaqaaKqbaoaalaaabaGaeiikaGIaemOCai3aaSbaaeaacqWGHbqyaeqaaiabcMcaPiabcIcaOiabigdaXiabgkHiTiabdkhaYnaaBaaabaGaemOyaigabeaacqGGPaqkaeaacqWGYbGCaaaaaaa@38D4@
sire^1^	dam^1^	(1−ra)(rb)r MathType@MTEF@5@5@+=feaafiart1ev1aaatCvAUfKttLearuWrP9MDH5MBPbIqV92AaeXatLxBI9gBaebbnrfifHhDYfgasaacPC6xNi=xH8viVGI8Gi=hEeeu0xXdbba9frFj0xb9qqpG0dXdb9aspeI8k8fiI+fsY=rqGqVepae9pg0db9vqaiVgFr0xfr=xfr=xc9adbaqaaeGacaGaaiaabeqaaeqabiWaaaGcbaqcfa4aaSaaaeaacqGGOaakcqaIXaqmcqGHsislcqWGYbGCdaWgaaqaaiabdggaHbqabaGaeiykaKIaeiikaGIaemOCai3aaSbaaeaacqWGIbGyaeqaaiabcMcaPaqaaiabdkhaYbaaaaa@38D3@

r : recombination probability from the Haldane mapping function between the two markers (r), between the left/right (r_a_/r_b_) marker and the QTL; ^1^: Probability of maternal origin is calculated analogously by inverting dam/sire origin of the markers.

*m *= *n*^2^*nzp*

where *n *is the number of individuals in the pedigree, *nz *is the proportion of non-zero elements in the matrix and *p *is a constant relating to the memory required to store two individual ID's as integers and a real valued IBD value with single (p ≈ 2 × 10^-5^) or double (p ≈ 2.9 × 10^-5^) precision.

**Figure 3:**
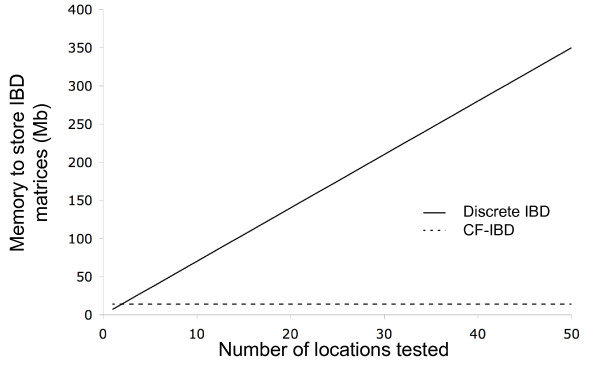
The required memory (in Mb) to store all IBD matrices in a marker bracket as individual matrices or as a single IBD function matrix for increasing numbers of tested locations in the bracket.

Storing a typical IBD-matrix in the chicken pedigree used in this study at single precision requires approximately *m *= 872^2 ^* 0.5 * 2 * 10^-5 ^≈ 7 Mb, as there are 872 individuals in the pedigree and around 50 % non-zero elements. If on the other hand the IBD functions for a marker interval are stored in the form of a sparse matrix that contains the three parameters (*a*, *b*, *c*) of a second degree polynomial (*a*x^2 ^+ *b*x + *c*) for each bracket and pair of individuals with single precision, then the constant *p *in [1] works out to be ≈ 4 × 10^-5 ^and the memory requirement to store one IBD-function matrix to about *m *= 872^2 ^* 0.5 * 4 * 10^-5 ^≈ 14 Mb. The advantage of this storage approach for varying number of IBD matrices in a marker bracket is illustrated in Figure 3.

Storing a large number of individual IBD-matrices representing an interval or and entire genome can thus require large amounts of memory if one wants to identify the QTL location with high precision. A single QTL genome scan with 1 cM resolution in the chicken genome requires approximately 2300 matrices corresponding to a memory requirement of 16 Gb, whereas the IBD-function based storage in a genome with on average 15 tested locations in a marker bracket (here corresponding to 15 cM marker spacing) requires about 2 Gb. In an epistatic analysis including two loci with a single interaction effect, the variance-covariance matrix for the interaction effect is usually constructed as the Hadamard product of the respective marginal effects IBD matrices [14]. An exhaustive genome-scan for two QTL (i.e. evaluating all potential pairs of loci for epistasis) involves accessing nearly 40 Tb of data for the single locus IBD matrices from disk unless the IBD matrices used to calculate interaction matrices are stored in RAM. This will increase the computational demand of the analyses considerably as the disk access time is in the order of milliseconds compared to that of nanoseconds when accessing RAM. The rather low and constant memory requirement posted by our new way to store and recall IBD matrices allows for storing all the necessary information in RAM, whereas the traditional approaches posts either large hardware requirements in terms of RAM (that increases dramatically with the desired resolution in the analyses) or long computational times due to excessive access of disk.

## Discussion

To facilitate development of methods for mapping multiple interacting QTL using variance component models, it is necessary to significantly improve the computational efficiency. To do so, three key components need to be addressed: estimation of relationship between relatives (i.e. IBD matrix estimation), variance component estimation of QTL at a given genomic location (or combination of locations) and the genome scan for genomic locations (or combinations of locations) to evaluate (i.e. the global optimization algorithm). Recently, more efficient algorithms for variance component estimation [9] and optimization methods for QTL genome scans [6,7] have been described. Here, we propose a new approach to describe IBD between individuals in the form of continuous marker bracket IBD functions. This improves memory usage and computational efficiency in the estimation of genome-wide IBD as well as facilitates implementation of existing and development of new and more efficient global optimization algorithm for detection of individual and multiple QTL.

We have shown that it is possible to estimate the IBD function for a marker bracket exactly by re-implementing existing IBD algorithms using Pong-Wong algorithm [10] as an example. This work illustrates that the IBD-function based approach generates the same IBD's and estimates of variance components as the original algorithm as well as a potential to improve both computational and memory usage. This approach should be applicable for most IBD estimation algorithms, although we have not shown this here, but will require an effort to be made for each particular IBD estimation algorithm in reformulating the computational algorithm and updating analysis software. This is the preferable strategy to obtain the optimal IBD functions and achieve maximal computational improvements by this approach.

In situations where re-implementation of the original algorithm is not achievable, but where IBD functions could be useful, we provide a general curve-fitting based algorithm to estimate these functions from IBD matrices provided by any existing algorithm. To illustrate the applicability of this approach, we have made an in-depth study of its properties. By using input from one IBD estimation method [3], we show that our approximation has a very small effect on the IBDs as well as on subsequently estimated QTL variance components. Moreover, a less detailed study with another IBD estimation method (Merlin; [4]) yielded similar results as the comparisons with LOKI (results not shown).

IBD probabilities can theoretically be calculated exactly, but in real datasets this will be computationally prohibitive. The methods used in practice thus aim to approximate the true IBD and depending on the assumptions made, methods will generally obtain different IBD matrices for a specific dataset. Here, we clearly show that differences in underlying assumptions in the algorithms may have a much greater impact on both the direct estimates if IBD's, as well as on variance component estimation, than from using IBD functions as approximations of a specific IBD estimation method.

By re-implementing an already fast deterministic IBD estimation algorithm by Pong-Wong et al [10] to conduct IBD-function estimation, it is possible to improve the computational efficiency in calculating IBD by a factor of 2 to 2.6 in most realistic situations. The algorithm used in the LOKI [3] software can simultaneously compute IBD estimates for any number of pre-defined locations in a marker bracket. Theoretically, our method to compute IBD functions from a limited set of IBD matrices does therefore not, decrease the computation-time of this method, but will decrease the memory requirement significantly. In practice, however, even though LOKI can theoretically compute any number of IBD matrices in a marker-bracket in a single run without significant additional computational cost, the limiting factor for performing a dense genome scan is the memory requirement to store a large number of IBD matrices across the genome. Thus, since in our work LOKI is not re-implemented to directly report the IBD functions for marker brackets, the IBD-function estimation approach will be useful to allow an increase in precision in the genome-scan, without an accompanying increase in memory requirement.

Our new approach facilitates the development of efficient algorithms for multi-dimensional genome scans for interacting QTL [15] by significantly reducing the hardware requirement for storing genome-wide IBD in RAM, which is a requirement for efficient analyses as storing and accessing large amount of data from disk would slow down the analyses significantly. This improved efficiency in the analysis makes analyses based on more advanced genetic modelling, including e.g. epistasis, more computationally tractable and thus of interest to a larger group of users.

The computational efficiency in QTL mapping in general can be improved considerably by replacing the commonly used grid search with a more efficient optimization algorithm [6,7]. Using this approach, the number of locations (or combination of locations) tested for QTL decreases and the computational efficiency is improved regardless of the IBD- or variance component estimation algorithms used. An inherent property of non-exhaustive grid searches is that the locations where IBD are needed cannot be predicted before the analysis. Thus, IBD either have to be computed on a pre-defined dense grid with high computational efficiency and high requirement for data storage or computed serially with a resulting decrease in the computational efficiency. By using the CF-IBD algorithm, however, it is possible to retain the computational efficiency in calculating IBD matrices in parallel, while minimizing the memory requirement in the analyses.

An existing optimization algorithm that would be interesting to explore for single- and multi-dimensional QTL searches in the variance component framework would be DIRECT [16]. This algorithm has been shown to be efficient in multi-dimensional QTL detection [7], and there greatly improves the computational efficiency. In addition to this, the availability of continuous IBD functions also opens up new opportunities to develop novel optimization algorithms for detecting QTL that explicitly uses the fact that there exists an underlying continuous function describing the genetic relationships between individuals across the genome.

## Conclusion

Here, we describe how and why to estimate IBD relationships in a population as a set of IBD functions between individuals. This approach immediately improves the efficiency in IBD matrix estimation in genome-scans for QTL, but also to facilitate further improvements by resolving methodological bottlenecks for implementing existing and developing new and more efficient algorithms to screen the genome for single or multiple QTL. We have provided two examples of how to estimate continuous IBD functions between a pair of markers, instead of a set of discrete IBD values, based on existing discrete IBD estimation algorithms. The first, and most efficient, method is a re-implementation of an existing IBD estimation algorithm to provide exact IBD functions that give the same values as the original algorithms. The second, more general method, approximates IBD-functions using IBD values from any algorithm of choice without the need for re-implementation of algorithms or software. The loss of information in this estimation is minor and it only affects QTL variance component estimates marginally. The estimation of continuous IBD-functions, rather than discrete IBD estimates, was shown to improve the computational efficiency (when re-implementing) and memory usage (for both strategies) in genome-scans for QTL. These improvements are expected to be of practical importance in particular in genome scans for multiple interacting QTL, where much higher computational and memory efficiency is required. The presented approaches will also has a positive impact on the use of more efficient search algorithms for QTL and the advent of continuous IBD functions opens new opportunities for developing new strategies for high-precision estimation of QTL location in single- and multidimensional genome scans.

## Methods

### Algorithms for genome-wide IBD estimation

#### Deterministic estimation of independent IBD matrices

**Figure 4:**
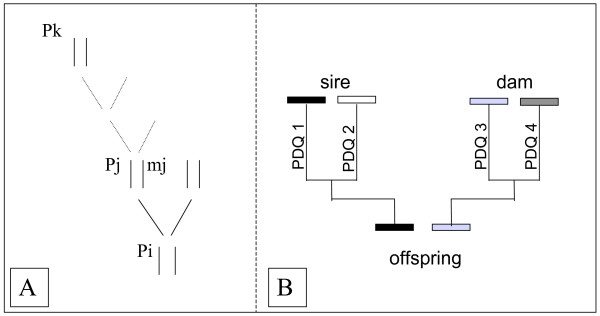
General schematic description of the calculation of Probability of Descent QTL (PDQ) of inheritance from ancestor to descendant (A) and from parent to offspring (B; PDQ1+PDQ2 = PDQ3+PDQ4 = 1) in the method by Pong-Wong et al [10].

Pong-Wong *et al *[10] describe an algorithm for recursive calculation of IBD matrices. We will summarize the principles of the method here to make the description of our updating of the algorithm to generate IBD-functions more transparent to the reader (Figure 4).

The original algorithm proceeds as follows. For each individual *i*, in a pedigree, paternally (*Pi*) and maternally (*Mi*) inherited chromosomes are assigned based on marker information and pedigree. IBD probabilities are calculated recursively for all chromosomes in the pedigree and summarized in an IBD-matrix. As individuals are diploids, the resulting matrix is of size *2n × 2n*, (rather than *n × n*), where *n *is the number of individuals in the pedigree.

Note that the deterministic algorithm estimates gametic IBD matrices (G) (of size 2n*2n), but the object that is used in the variance component method is an individual matrix (of size n*n), where the four element of G, corresponding to IBD between the four chromosomes of a pair individual, are summed up to obtain a single IBD value between the two individual.

In the result part, when comparing values given by the different IBD estimation methods, we always refer to the individual IBD matrix.

Considering an individual *i*, his father *j *and a given ancestor *k*, The IBD probability between the paternal chromosome, (*P*_*i*_), of individual *i*, and the chromosome *P*_*k *_of the ancestor *k*, is equal to:

IBD(*P*_*i*_,*P*_*k*_) = IBD(*P*_*k*_,*P*_*j*_)*PDQ(*P*_*i*_,*P*_*j*_) + IBD(*P*_*k*_,*M*_*j*_)*PDQ(*P*_*i*_,*M*_*j*_)

(Figure 4a)

where PDQ (Probability Descent QTL) is the probability that a particular locus of a chromosome (*P*_*i*_) from the parent *j *is inherited either from *j*'s paternal chromosome (*P*_*j*_) or *j*'s maternal chromosome (*M*_*j*_). Consequently, the sum of the PDQ for the two parental chromosomes is equal to one (Figure 4b). Each PDQ is calculated as a function of the distance of the considered locus from the two flanking markers, expressed as a recombination probability [10], and the algorithm for the calculation of an IBD matrix at the genomic location *g *consists of the following steps:

*i*) Search for the closest informative markers flanking *g*

*ii*) Determine which allele (paternal or maternal) that have been inherited for each of the two flanking markers

*iii*) For each pair of individuals in the pedigree that have a parent-offspring relationship, calculate the PDQs at *g*, using a pre-specified recombination probability, *r*, between the markers that is estimated from the genetic map.

*iv*) For each pair of chromosomes, from ancestor to descendant, calculate the IBD values recursively.

**Figure 5:**
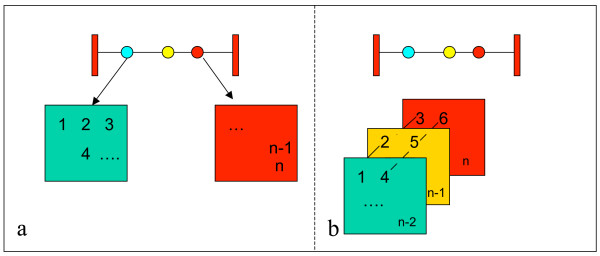
Schematic description of independent (A) and horizontal (B) calculation of IBD matrices at genomic locations in intervals between genetic markers.

Since IBD within the same bracket are considered independently, steps i)-iv) are performed successively each time an IBD matrix is calculated. (Figure 5a)

#### Updated deterministic algorithm for estimation of marker bracket IBD functions

Existing methods for estimating IBD matrices consider IBD values in the same bracket to be independent. However, IBDs within a marker bracket are not independent and this fact can be used to simultaneously infer all IBD values in a bracket. An updated version of the algorithm of Pong Wong *et al*. [10], that do this proceeds as follows:

a) Steps *i) *and *ii) *of the original algorithm are completed once for a marker bracket. When the parental inheritance has been inferred in step ii), one can select the recombination functions for calculation PDQs between pairs of individuals. This function is the same for all positions between the markers. We give these functions in Table 1.

b) For all parent-offspring pairs, PDQs are computed "horizontally" for each pair of chromosomes (Figure 5b), using the original recursive strategy [10]. With horizontally we mean that PDQs are calculated at all positions within the bracket before the algorithm proceeds to the next pair of chromosomes. This is possible since, within a bracket, the PDQs for a given pair of chromosomes are given by the same recombination-probability function; only the position, *g*, within the marker bracket (represented by *ra *and *rb *in the formulas in Table 1) changes.

c) For all pairs of individuals in the pedigree, IBD probabilities are computed horizontally, for all positions between the markers, using the Pong-Wong original recursive strategy illustrated in Figure 5b.

The updated Pong-Wong algorithm thus computes several IBD matrices simultaneously, which improves the computational efficiency by minimizing the computational cost of reading data and searching for informative markers.

#### Stochastic IBD-matrix estimation

LOKI [3] is a freely available linkage analysis package. It can also be used for estimation of IBD matrices in general pedigrees using a Markov Chain Monte Carlo (MCMC) based algorithm. As MCMC is an iterative method, the user has to select the number of required iterations by the program. With our data, the output matrices given by LOKI after 10,000 iterations were unstable (i.e. the IBD probabilities varied considerably from one run to another). 100,000 iterations provided stable estimates and was therefore used throughout our study.

#### A general algorithm for estimation of marker bracket IBD functions

We propose a general algorithm for estimation of marker bracket IBD functions that as input use IBD values at marker locations calculated using an external IBD estimation algorithm suitable for the experimental data that is analysed. Using information from the IBD-matrices at the markers, a continuous function of IBDs is estimated in the brackets between markers. This approach is possible as the IBD values within a marker bracket are a continuous function of their location in the bracket and strongly correlated with the IBD values at the flanking marker positions. From the estimated set of IBD functions in the pedigree, IBD matrices can then be directly computed at any location between the markers at a very low computational cost.

IBD-functions for each pair of individuals in a marker bracket can be estimated using curve fitting based on the functions of Table 1, which represent the recombination probability between linked markers for the base relationships in the pedigree. Each of the recombination probabilities in Table 1 can be approximated numerically by a second-degree polynomial function. This makes it straightforward to estimate a polynomial function that fits the values given by LOKI at marker location.

The algorithm can be summarized as follows: For each pair of individuals, the IBD values at the three positions: two flanking markers (IBD(*LM*); IBD(*RM*)) plus one position between the parkers (IBD(*M*) are calculated using any algorithm of choice (e.g. LOKI). Expressed in terms of the distance between the two makers (*d*), the position between the two markers where an IBD will be calculated (*x*) and setting M = *d*/2 we have:

IBD(*x*) = IBD(*LM*) when *x *= 0

IBD(*x*) = IBD(*M*) when *x *= *M*

IBD(*x*) = IBD(*RM*) when *x *= *d*

A polynomial function (IBD(*x*) = ax^2 ^+ bx + c) can then be fitted by solving the equation system:

{c=IBD(LM)a(d2)2+12bd+c=IBD(M)ad2+bd+c=IBD(RM)
 MathType@MTEF@5@5@+=feaafiart1ev1aaatCvAUfKttLearuWrP9MDH5MBPbIqV92AaeXatLxBI9gBaebbnrfifHhDYfgasaacPC6xNi=xI8qiVKYPFjYdHaVhbbf9v8qqaqFr0xc9vqFj0dXdbba91qpepeI8k8fiI+fsY=rqGqVepae9pg0db9vqaiVgFr0xfr=xfr=xc9adbaqaaeGacaGaaiaabeqaaeqabiWaaaGcbaWaaiqabeaafaqaaeWabaaabaGaem4yamMaemypa0JaemysaKKaemOqaiKaemiraqKaeiikaGIaemitaWKaemyta0KaeiykaKcabaGaemyyae2aaeWaaKqbagaadaWcaaqaaiabdsgaKbqaaiabbkdaYaaaaOGaayjkaiaawMcaamaaCaaaleqabaGaeeOmaidaaOGaem4kaSscfa4aaSaaaeaacqqGXaqmaeaacqqGYaGmaaGccqWGIbGycqWGKbazcqWGRaWkcqWGJbWycqWG9aqpcqWGjbqscqWGcbGqcqWGebarcqGGOaakcqWGnbqtcqGGPaqkaeaacqWGHbqycqWGKbazdaahaaWcbeqaaiabdkdaYaaakiabdUcaRiabdkgaIjabdsgaKjabdUcaRiabdogaJjabd2da9iabdMeajjabdkeacjabdseaejabcIcaOiabdkfasjabd2eanjabcMcaPaaaaiaawUhaaaaa@5E86@

which gives:

{c=IBD(LM)b=4IBD(M)−3IBD(LM)−IBD(RM)da=2IBD(RM)+2IBD(LM)−4IBD(M)d2
 MathType@MTEF@5@5@+=feaafiart1ev1aaatCvAUfKttLearuWrP9MDH5MBPbIqV92AaeXatLxBI9gBaebbnrfifHhDYfgasaacPC6xNi=xI8qiVKYPFjYdHaVhbbf9v8qqaqFr0xc9vqFj0dXdbba91qpepeI8k8fiI+fsY=rqGqVepae9pg0db9vqaiVgFr0xfr=xfr=xc9adbaqaaeGacaGaaiaabeqaaeqabiWaaaGcbaWaaiqabeaafaqaaeWabaaabaGaem4yamMaemypa0JaemysaKKaemOqaiKaemiraqKaeiikaGIaemitaWKaemyta0KaeiykaKcabaGaemOyaiMaemypa0tcfa4aaSaaaeaacqqG0aancqWGjbqscqWGcbGqcqWGebarcqGGOaakcqWGnbqtcqGGPaqkcqGHsislcqqGZaWmcqWGjbqscqWGcbGqcqWGebarcqGGOaakcqWGmbatcqWGnbqtcqGGPaqkcqGHsislcqWGjbqscqWGcbGqcqWGebarcqGGOaakcqWGsbGucqWGnbqtcqGGPaqkaeaacqWGKbazaaaakeaacqWGHbqycqWG9aqpjuaGdaWcaaqaaiabbkdaYiabdMeajjabdkeacjabdseaejabcIcaOiabdkfasjabd2eanjabcMcaPiabdUcaRiabbkdaYiabdMeajjabdkeacjabdseaejabcIcaOiabdYeamjabd2eanjabcMcaPiabgkHiTiabbsda0iabdMeajjabdkeacjabdseaejabcIcaOiabd2eanjabcMcaPaqaaiabdsgaKnaaCaaabeqaaiabbkdaYaaaaaaaaaGccaGL7baaaaa@719F@

**Figure 6:**
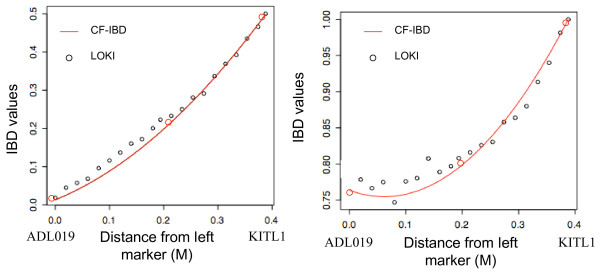
Estimation of a polynomial IBD function for a pair of individuals in a marker bracket using IBDs at the right and left flanking marker and at the mid-point in the interval as input.

Polynomial functions representing the IBD relationship for each pair of individuals in the pedigree are then estimated as illustrated in Figure 6.

### Variance component estimation

An AI-REML algorithm [17] programmed in R was used to estimate the additive QTL variance component for a given IBD matrix (L.Rönnegård personal communication).

## Authors' contributions

ÖC initiated the study. ÖC and FB designed and developed the project and wrote the paper together. All authors have read and approved the manuscript.
